# Examination of morphological traits of children's faces related to perceptions of cuteness using Gaussian process ordinal regression

**DOI:** 10.3389/fpsyg.2022.979341

**Published:** 2022-11-03

**Authors:** Masashi Komori, Teppei Teraji, Keito Shiroshita, Hiroshi Nittono

**Affiliations:** ^1^Graduate School of Engineering, Osaka Electro-Communication University, Neyagawa, Japan; ^2^Graduate School of Human Sciences, Osaka University, Suita, Japan

**Keywords:** Gaussian process ordinal regression, Bayesian optimization, face, baby schema, cuteness

## Abstract

Konrad Lorenz, an ethologist, proposed that certain physical elements are perceived as cute and induce caretaking behavior in other individuals, with the evolutionary function of enhancing offspring survival. He called these features Kindchenschema, baby schema. According to his introspection, these include a large forehead, chubby round features, and chubby cheeks. Previous studies are limited to examining the effects of these facial features on perceived cuteness. However, other morphological factors may be related to perceived cuteness. This study uses Bayesian optimization, one of the global sequential optimization methods for estimating unknown functions, to search for facial morphological features that enhance the perceptions of facial cuteness. We applied Bayesian optimization incorporating Gaussian process ordinal regression (GPOR), which allows an estimation of the latent cuteness function based on evaluations using the Likert scale. A total of 96 preschool children provided the facial images used in this study. We summarized the facial shape variations using methodologies of geometric morphometrics and principal component analysis (PCA) up to the third principal component (PC), which we refer to as the face space. A total of 40 participants evaluated the images created by warping the average facial texture of the children's faces with randomly generated parameters in the face space. Facial traits related to perceived cuteness were estimated based on the averaged cuteness function. Perceived cuteness was linked to the relative lower position of facial components and narrower jawline but not to the forehead height.

## 1. Introduction

Ethologist Konrad Lorenz pointed out that certain physical features of animals induce specific behaviors and affective responses related to caretaking behaviors (Lorenz, 1943). Further, he called such universal features across species the baby schema (“Kindchenschema”). Lorenz argued that baby schema includes features such as a large head, big eyes, high and protruding forehead, chubby cheeks, small nose, and mouth, that are perceived as cute. Many empirical studies have supported this idea, suggesting a biological and evolutionary basis for cognitive and emotional responses to specific features typical of children's faces (Kringelbach et al., 2016). For example, some studies have shown that infants' level of facial cuteness is associated with a large forehead, small chin, full lips, and chubby round features (Hildebrandt and Fitzgerald, 1979; Almanza-Sepúlveda et al., 2018) suggesting that faces that are perceived as cute have some traits in common.

However, facial traits that elicits the perception of cuteness are not identical to the facial features possessed by human infants. This is because the emphasis on infant facial traits does not necessarily increase the levels of perceived cuteness of children's faces (Komori and Nittono, 2013). Therefore, the factors that induce the perception of cuteness cannot be clarified only by examining the objective characteristics of human infants. Thus far, different approaches have been used to investigate which facial features affect the perception of cuteness. One approach is to take specific facial elements, such as roundness of the face, the height of forehead and eyes, nose and mouth size, and manipulate the images directly to examine their effects on perceived cuteness (Sternglanz et al., 1977; Alley, 1981; Glocker et al., 2009; Borgi et al., 2014; Endendijk et al., 2018; Löwenbrück and Hess, 2021). However, there is a limit to this method of determining *a priori* which features to manipulate since there may be unknown factors that increase the levels of cuteness. Another approach is to create facial images having features that enhance cuteness by computationally compositing images of faces with high cuteness (Sprengelmeyer et al., 2009; Lobmaier et al., 2010; Hahn et al., 2013; Nittono et al., 2022). However, such a method cannot clarify which features are involved in cuteness perception and the degree to which they are involved. The other approach is analyzing the correlation between the locations of facial landmarks and perceived cuteness. Almanza-Sepúlveda et al. (2018) examined the relationship between infants' facial traits and cuteness based on a linear regression analysis. However, it is possible that the relationship between facial traits and cuteness is non-linear, similar to that between facial traits and attractiveness (Komori et al., 2009b). Thus, this study aims to solve these problems of previous methodologies by using a method that can comprehensively examine the non-linear relationship between morphological facial traits and perceived cuteness.

We assume that people have their own psychological function *f*(**x**) (the present study refers to this function as the utility function) that maps the multivariate input **x** (i.e., certain traits of a presented face) to a scalar value representing the degree of perceived cuteness of the person whose face has been assessed. Thus, the problem of elucidating the impression evaluation mechanism for others' faces can be regarded as the problem of estimating the parameters of the utility function *f*(**x**).

The input **x** of the utility function corresponds to the morphological features of the faces. Instead of arbitrarily targeting specific facial features for investigation, such as forehead size and eye position, this study uses a methodology of geometric morphometrics (Bookstein, 1991; Dryden and Mardia, 1998) to represent the features of infants' facial shapes in a few dimensions. Geometric morphometrics is a technique used to represent facial shape as multivariate data (Komori et al., 2009b).

Even if facial features are represented in a small number of dimensions, the number of combinations is so large that it is not easy to reveal the utility function *f*. One way to thoroughly examine a psychological utility function *f*(**x**) is to explore the search space via a grid search. However, it is challenging to obtain responses to multidimensional stimuli by performing a grid search because of the high cost of the evaluation task. Moreover, since the utility function of cuteness perception may be complicated, it is inappropriate to apply methods, such as conjoint analysis, which does not consider feature combinations, to study the cuteness perception. Since the utility function of perceived cuteness may be multi-peaked or have a complex shape, it is also inappropriate to apply methods such as conjoint analysis (Rao et al., 2014), which does not take into account feature combinations, to the study of cuteness perception.

This study aimed to estimate the relationship between child facial features and perceived cuteness using the Gaussian process regression (GPR) model (O'Hagan, 1978; Neal, 1997), a non-parametric Bayesian approach to metric regression. Gaussian process regression is a method for estimating an unknown black-box function using a kernel function and is often used to find the maximum/minimum value of the function. Unlike linear regression analysis, GPR has the advantage of being able to estimate non-linear functions, such as multi-peaked functions, and also provide probabilistic predictions.

However, there are problems in using GPR to estimate psychological utility functions. In a typical GPR method, the return value (i.e., the responses from the participants) from an unknown function is expected to be a continuous quantity. Conversely, psychological research commonly uses methods that require discrete responses from participants, such as Likert scales, dichotomous choices, and paired comparison methods. Therefore, using discrete responses to estimate psychophysical functions based on natural human responses is desirable.

We used the Gaussian process ordinal regression (GPOR) (Chu et al., 2005), which can estimate non-linear function *f* from discrete responses (i.e., responses to a Likert scale).

Let $D=\left({\text{x}}_{i},y_{i}\right)|i=1,\ldots ,N=\left(\text{x},\text{y}\right)$ denote the set of facial traits **x**_*i*_ and the corresponding cuteness evaluation label *y*_*i*_ ∈ *L*, where *L* is a finite set of *R* ordinal categories, denoted *L* = {1, 2, …*R*}. We assume that the utility function *f* follows a zero mean Gaussian process prior defined by a kernel function K.


(1) $$\begin{array}{l} f\sim \mathrm{GP}\left(0,K\right) \end{array}$$

Here, we used an RBF ARD kernel (MacKay, 1996).


(2) $$\begin{array}{l} K\left({\text{x}}_{i},{\text{x}}_{j}\right)=\exp\left(-\frac{1}{2}\overset{\text{D}}{\underset{d=1}{\sum }}\eta_{d}{\left(x_{di}-x_{dj}\right)}^{2}\right) \end{array}$$

Let **b** be the threshold variable, where *b*_0_ < *b*_1_ < ⋯ < *b*_*r*_ and *b*_0_ = −∞, *b*_*r*_ = ∞, which map *f*(*x*_*i*_) to the discrete variable *y*_*i*_. Under noise-free conditions, the ideal likelihood function would be defined as $P_{ideal}\left(y_{i}|f\left({\text{x}}_{i}\right)\right)=1$ when *b*_*y*_*i*_−1_ < *f*(**x**_*i*_) < *b*_*y*_*i*__ (0 otherwise). In the presence of noise from inputs or targets, the latent functions are contaminated by Gaussian noise with zero mean and unknown variance, denoted $N\left(\delta ;0,\sigma^{2}\right)$, where δ is a Gaussian random variable. The ordinal likelihood function becomes


(3) $$\begin{array}{l} P\left(y_{i}|f\left(x_{i}\right)\right)=\Phi \left(z_{i}^{\left(y_{i}\right)}\right)-\Phi \left(z_{i}^{\left(y_{i}-1\right)}\right) \end{array}$$

(4) $$\begin{array}{l} z_{i}^{\left(s\right)}=\frac{b_{s}-f\left(x_{i}\right)}{\sigma_{\delta }} \end{array}$$

where Φ(*z*) is the cumulative unit Gaussian (CDF) whereby $\Phi \left(z\right)=\int_{-\infty }^{z}N\left(\gamma ;0,1\right)d\gamma$. Based on Bayes' theorem, the posterior probability can then be written as


(5) $$\begin{array}{l} P\left(f|D\right)=\frac{1}{P\left(D\right)}\overset{n}{\underset{\left(i=1\right)}{\prod }}P\left(y_{i}|f\left(x_{i}\right)\right)P\left(f\right) \end{array}$$

To approximate the posterior distribution and model evidence P(D) we used the Laplace approximation at the maximum a posteriori (MAP) estimate (Williams and Barber, 1998). Under the Laplace approximation, the predictive distribution of the utility function can be described as a Gaussian $N\left(f\left(x\right);\mu ,\sigma^{2}\right)$ where the predictive mean (μ_x*_) and variance ($\sigma_{x^{*}}^{2}$) for which the response y_*_ is unknown.


(6) $$\begin{array}{l} \mu_{x}*=k{*}^{T}K^{-1}f_{MAP} \end{array}$$

(7) $$\begin{array}{l} \sigma_{x*}^{2}=K(X,X)-k_{*}^{T}{\left(K+\Lambda_{MAP}^{-1}\right)}^{-1}k_{*} \end{array}$$

where; k_*_ is the covariance between the test case and the training data. Further, *f*_**MAP**_ is the MAP estimate of the utility function, **Λ**_**MAP**_ is a diagonal matrix whose *i*-th entry is the second derivative of the likelihood function training sample *i* concerning *f*(**x**_*i*_).

This study examines the effectiveness of GPOR as a novel method for revealing the utility function *f*, a black box function that describes the relationship between multidimensional facial features and perceived cuteness, from two perspectives.

First, we compare the predictive performance of the GPOR model used in this study with an ordinal logistic regression model, a standard generalized linear regression model for ordinal data, using the leave-one-out cross-validation (LOOCV) approach.

Second, we average the utility functions obtained from the participants in the experiment and examine their shape. Furthermore, by identifying the face shapes corresponding to the maximum and minimum values of the average utility function, we explore the factors that determine the perceived facial cuteness of a child. Finally, these results will be discussed based on whether GPOR can provide useful information in psychological studies.

## 2. Computational analysis of facial images

### 2.1. Materials and facial shape measurement

Japanese preschool children (*n* = 96, 48 boys and 48 girls; age 3–4 years, mean age = 3.98, SD = 0.55) provided the facial frontal images used in this study. Written informed consents were obtained from the legal guardians for the publication of non-personally identifiable images or data included in this article. A neutral expression on each face was captured using a digital camera. Additionally, the foreheads of the models were exposed using a headband, after removing head accessories such as eyeglasses. These images are the same as those used in the authors' previous study (Komori and Nittono, 2013).

Eighty facial landmarks were selected based on a previous study (Komori et al., 2009a). These landmarks consisted of morphological and functional points, such as the pupils, contours of eyes, eyebrows, nose, and mouth, among others. The authors visually measured all landmarks for each of the 96 photographs using a program written by the authors.

### 2.2. Facial shape standardization

Each face differed in location, size, and orientation. To standardize them, we performed a generalized Procrustes analysis (GPA) on the facial landmarks of all faces. This method preserves information about the relative spatial relationships of landmarks throughout the standardization. For the standardization of location and size, we used the centroid size technique (Bookstein, 1991). All facial shapes were translated for that the centroid (center of gravity) to have the exact location scaled to the same centroid size, which is the sum of the squared distances from the centroid to each landmark. For alignment of the orientation, rotations around the centroid of the faces (Dryden and Mardia, 1998) were performed to minimize the sum of the squared distances among corresponding feature points between samples. The “Shapes” statistical package, which runs in an R statistical analysis environment, was employed for the analyses.

Facial differences derived from facial asymmetries are not the focus of this study. Therefore, to exclude facial variations derived from facial asymmetries and the coordinates of original facial images, the mirror-reversed versions of the same images were used to create a “symmetrical version” of the individuals following the procedures of a previous study (Komori et al., 2009a). Consequently, each “symmetrical version” of the individuals was represented as a point on a linear space of 160 dimensions (x- and y-coordinates × 80 landmarks). This study refers to this multidimensional space as “face space.” The center of this face space corresponds to the average face of the “symmetrical version” faces.

**Figure 1:**
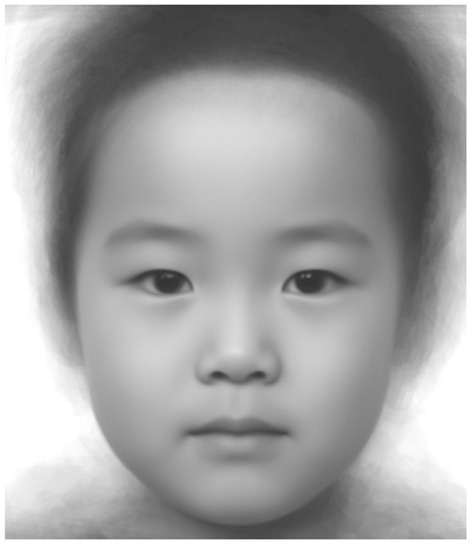
Average facial image.

All face images were gray-scaled and warped to the average face landmark coordinates using thin-plate splines transformation (TPS), a non-linear image deformation technique. Then, the average face texture was synthesized by averaging the luminance values of the warped images (Figure 1).

### 2.3. Facial feature extraction

**Figure 2:**
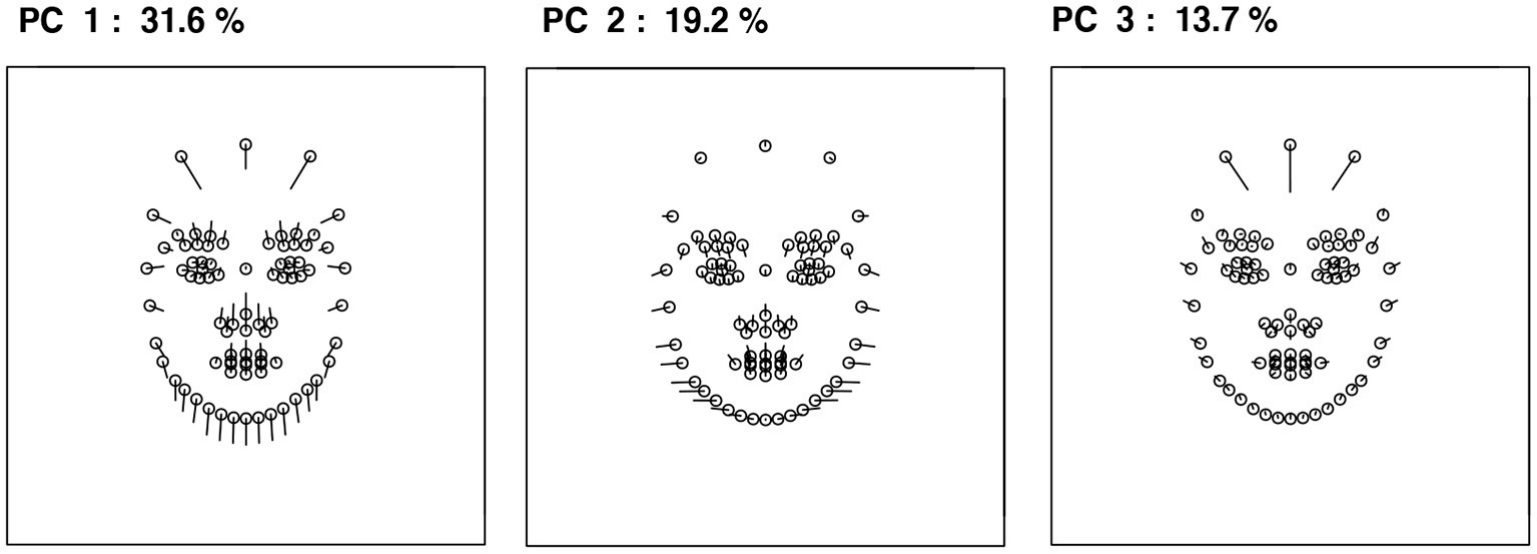
Facial shape changes along each PC. When the score of each PC increases, each facial landmark (depicted as a circle) moves along the leading line.

Variations in children's facial features were summarized using a principal component analysis (PCA). The results of the PCA indicated that the contributions of the first three principal components (PCs) for the total variance were relatively large (PC1: 31.6%; PC2: 19.2%; PC3: 13.7%; cumulative contribution ratio: 64.5%), and thus up to the PC3 was considered in the study. Based on the distributions of children's facial shapes, the facial landmark coordinates changes along each PC were calculated (Figure 2). The PC1 was related to lower chin position and lower hairline, indicating that PC1 is related to relative eye and mouth position. Further, the higher the score, the higher the relative eye and mouth position. The PC2 was associated with the face width face from the cheekbones to the jaw. PC3 was linked to the height or the length of the forehead.

**Figure 3:**
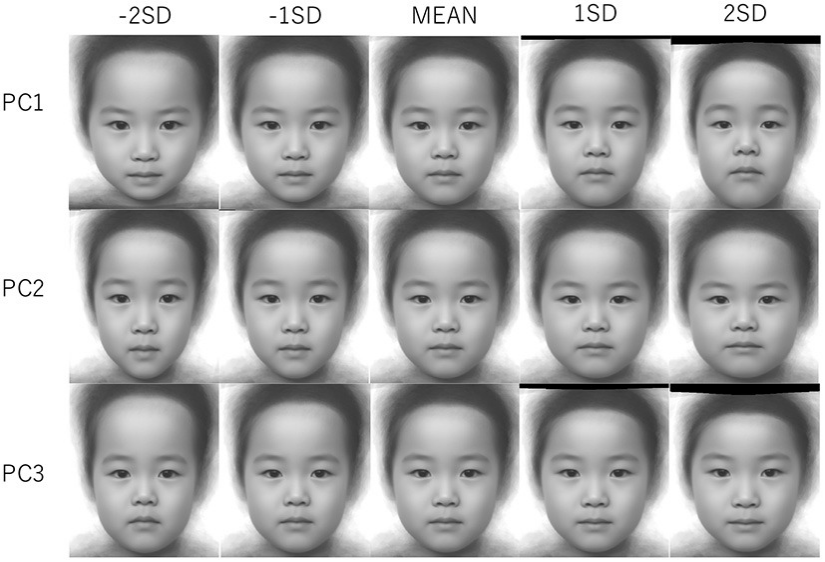
Differences in face shape along the each principal component (−2 SD/−SD/MEAN/+SD/+2 SD). The aspect ratios of the images were different from each other due to TPS warping.

The facial images corresponding to the theoretical values of −2SD, −SD, average, +SD, and +2SD along each PC were made by warping the average facial texture of the children's faces using TPS (Figure 3).

## 3. Assessment of perceived cuteness

### 3.1. Participant

Forty undergraduates (20 men and 20 women; mean age = 22.13, SD = 2.20) participated in the assessment of facial cuteness.

### 3.2. Procedure

The images presented to the participants are the warped images of the average facial image (Figure 3) using TPS so that the facial shapes correspond to given PC scores in the face space. Participants were instructed to evaluate the levels of cuteness of face images. Further, they were rated on a 5-point scale where 1 = not cute (*kawaii* in Japanese) at all and 5 = very cute ((*kawaii*) according to their first impressions of the images, i.e., without contemplating their responses. No time limit was set for the responses in each trial. Facial images were presented on an LCD monitor. The participants responded with a keyboard.

The rating task consists of 20 practice trials and 60 test trials.

In the first 45 test trials, the stimulus images were generated from the PC scores randomly selected from a range of ±2SD in the face space. In the subsequent 15 trials, the images were generated from the PC scores at which the upper confidence bound (UCB) values are maximized based on the response history of the participants in the previous test trials where *N* is the number of trials:


(8) $$\begin{array}{l} {\text{UCB}}_{\text{x}}=\mu_{\text{x}}+\sqrt{\frac{\log N}{N}}\sigma_{\text{x}}. \end{array}$$

Upper confidence bound is a type of acquisition function often used in Bayesian optimization. This type of experimental procedure is called a sequential experimental design. The trials were separated by 5 s of a blank gray screen. Each participant completed the rating task in about 30 min. The application used in the experiments was implemented in a PsychoPy environment (Peirce, 2007).

## 4. Results

### 4.1. Leave-one-out error analysis

The predictive performance of GPOR was examined using leave-one-out cross-validation (LOOCV). Specifically, we withheld one response from each participant. Further, we fit the models of GPOR and ordinal logistic regression on the remaining responses of the participant and formed a prediction of the held-out response using each of the learned models. We then compared each learned model's predictions with the observed responses for the held-out trial and computed the zero-one error and the squared error. Zero-one error gives an error of 1 to every incorrect prediction. Absolute error is the deviation of the prediction from the actual target. By computing the mean of the held-out zero-one errors (MZE) and the mean of absolute errors (MAE) across all participants, we can evaluate the predictive performance of the models.

- Mean zero-one error (MZE): the fraction of incorrect predictions on test data; $\frac{1}{N}\overset{N}{\underset{i=1}{\sum }}I\left({ŷ}_{i}\neq y_{i}\right)$, where *I*(·) denotes an indicator function which gives 1 when the argument is true and 0 otherwise.
- Mean absolute error (MAE): the average deviation of predicted test outputs from the true rank, in which we treat the ordinal scales as consecutive integers, $\frac{1}{N}\overset{N}{\underset{i=1}{\sum }}|{ŷ}_{i}-y_{i}|$.

**Figure 4:**
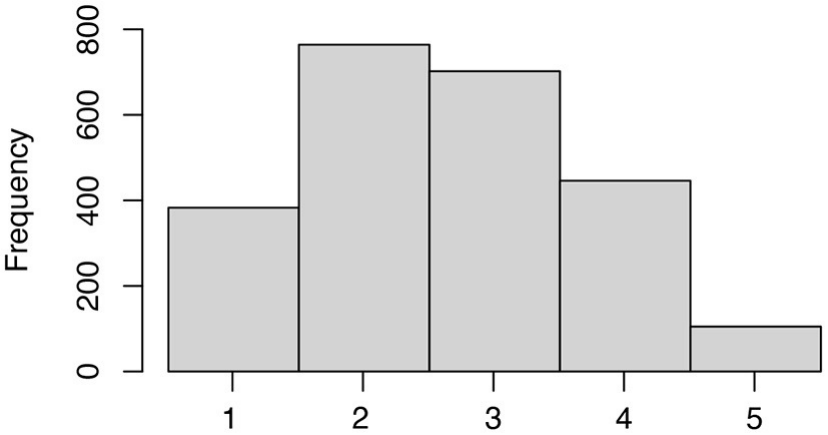
Distribution of ratings.

The distribution of ratings is shown in Figure 4. The MZE and mean MAE of the predictions by the GPOR model and by ordinal logistic regression were obtained by LOOCV (Table 1).

**Table 1:** Cross-validation results.

	**MZE**		**MAE**	
	**Test**	**Train**	**Test**	**Train**
Ordinal logistic regression	0.723	0.664	1.122	0.942
GPOR	0.655	0.455	0.82	0.525

Mean zero-one error refers to the fraction of classes predicted that differ from the correct response, which is the fraction of incorrect predictions. This study's chance level of MZE is 0.8 because the participants were given a 5-point scale. The MZEs of both models had higher prediction accuracies than chance level [ordinal logistic regression: *t*_(39)_ = −4.71, *p* < 0.001, Cohen's *d* = 0.74; GPOR: *t*_(39)_ = −6.71, *p* < 0.001, Cohen's *d* = 1.06]. The GPOR model had a lower error rate than the ordinal logistic regression model [*t*_(39)_ = −2.81, *p* = 0.007, Cohen's *d* = 0.44]. Mean of absolute error is the average deviation of the prediction from the actual target values in which ordinal scales are treated as consecutive integers, and, as with MZE, GPOR performed better than ordinal logistic regression [*t*_(39)_ = −5.87, *p* < 0.001, Cohen's *d* = 0.93]. These results suggest that GPOR is more effective than the generalized linear model in estimating the utility function that represents the relationship between facial features and perceived cuteness.

### 4.2. Facial shape that maximizes and minimizes perceived cuteness

The utility function *f*_*s*_ of each participant *s* is expressed as a set of predicted means estimated by using GPOR. The predicted means and the variances were obtained within ±2*SD*. of each dimension of the PCs with an interval of 0.4, resulting in 1,331 points per participant. The maximum value of utility function $f_{s}\left({\text{x}}_{s}^{*}\right)$ for each participant was shown to be significantly greater than zero [$p\left(f_{s}\left({\text{x}}_{s}^{*}\right)\leq 0\right)<0.01$ for each participant], where ${\text{x}}_{s}^{*}=\arg\max_{\text{x}\in A}f_{s}\left(\text{x}\right)$ in face space *A*, indicating that differences in face shape had a consistent influence on individual judgments.

Next, we calculated the mean of the predicted mean μ_*S***x**_ and the predicted variance $\sigma_{S\text{x}}^{2}$ for each coordinate **x**. *M* denotes the number of participants in this study.


(9) $$\begin{array}{l} \mu_{S\text{x}}=\frac{1}{M}\overset{M}{\underset{s=1}{\sum }}\mu_{s\text{x}} \end{array}$$

(10) $$\begin{array}{l} \sigma_{S\text{x}}^{2}=\frac{1}{M}\overset{M}{\underset{s=1}{\sum }}\left(\mu_{s\text{x}}^{2}+\sigma_{s\text{x}}^{2}\right)-\mu_{S\text{x}}^{2} \end{array}$$

**Figure 5:**
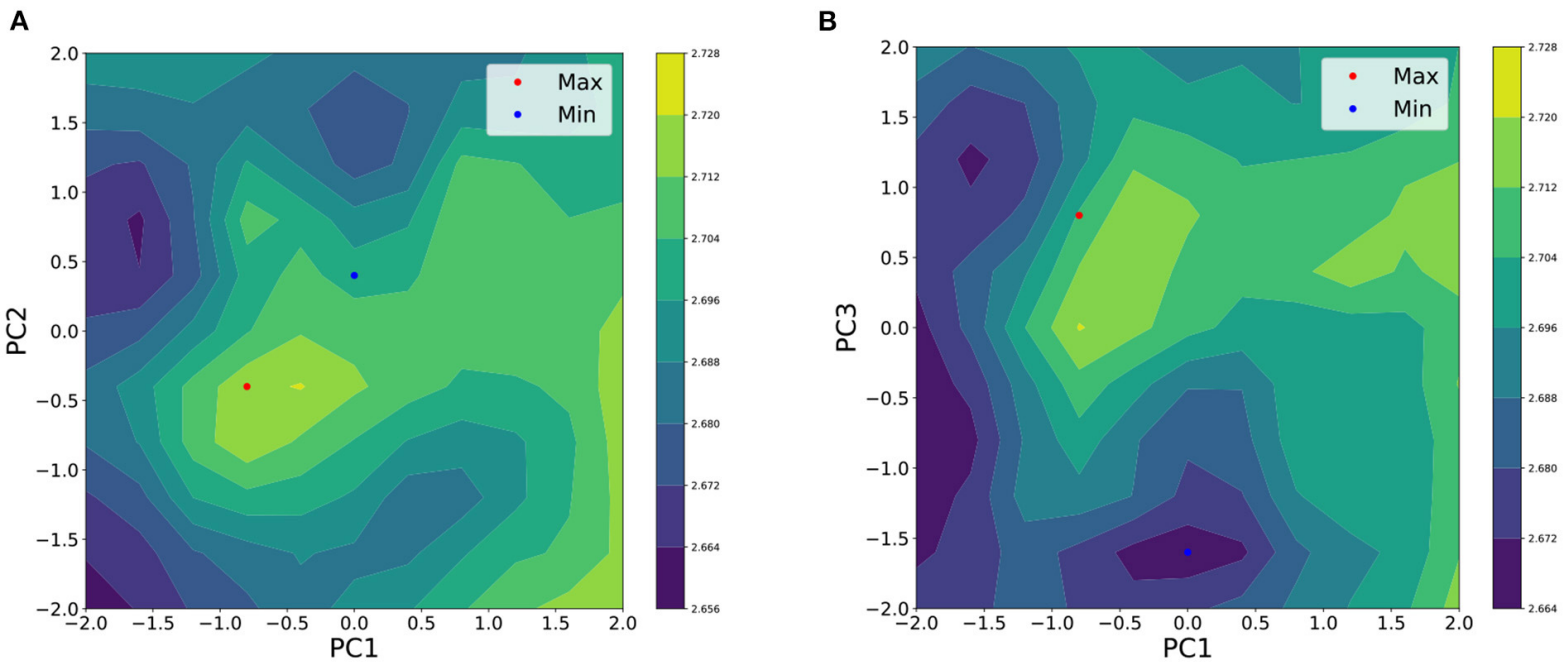
Averaged utility function of perceived cuteness on **(A)** PC1 and PC2 and **(B)** PC1 and PC3. The height of each surface describes the strength of the perceived cuteness. Red dots represent the PC score corresponding to the maximum of the function and blue dots represent the PC scores corresponding to the minimum.

Here, μ_*S***x**_ is an estimate of the average perceived cuteness for a given facial feature **x**, and we refer to the set of μ_*S***x**_ as the average utility function *f*_*S*_. Figure 5 the relationship between the first, second, and third PCs of facial traits and the average perceived cuteness. The perceived cuteness is higher for the combination of slightly lower the first and the second PC scores. However, the high scores of the third PC are consistently associated with perceived cuteness.

x^*^ denotes the coordinate of a maximum of averaged cuteness in the face space *A*.


(11) $$\begin{array}{l} {\text{x}}^{*}=\arg\underset{\text{x}\in A}{\max}\mu_{S\text{x}} \end{array}$$

The maximum predicted mean $\mu_{S{\text{x}}^{*}}$ of the function *f*_*S*_ was significantly greater than zero $\left[p\left(f_{S}\left({\text{x}}^{*}\right)\leq 0\right)<.05\right]$, according to the combined predicted variance $\sigma_{Sx^{*}}^{2}$. This suggests that averaging utility functions of the participants is valid in examining the average tendency of judgments.

The coordinates of the maximum value x^*^ of the average utility function were obtained (PC1: −0.8, PC2: −0.4, PC3: 0.8). Further, the coordinates of minimal value **x**^−^ of the average utility function were obtained (PC1: 0.0, PC2: 0.4, PC3: −1.6).


(12) $$\begin{array}{l} {\text{x}}^{-}=\arg\underset{\text{x}\in A}{\min}\mu_{S\text{x}} \end{array}$$

**Figure 6:**
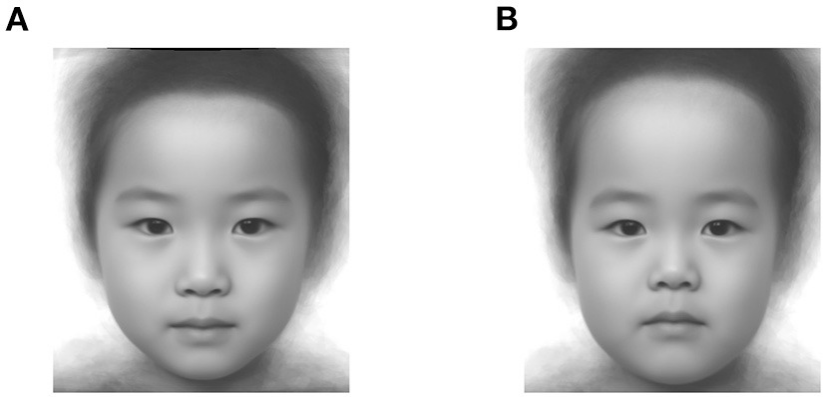
Facial images generated from the parameters corresponding to **(A)** the maximum (PC1: -0.8, PC2: -0.4, PC3: 0.8) and **(B)** the minimum of the averaged utility function of cuteness (PC1: 0.0, PC2: 0.4, PC3: −1.6).

Figure 6 shows the face images corresponding to the maximum and minimum values of the average utility function.

The face perceived to be the cutest, on average, is characterized by low eye position, smaller chin, narrow jawline and forehead, according to the first PC related to relative eye position, the second to jawline width, and the third to forehead height. Conversely, the face with the minimum utility function for perceived cuteness is characterized by high eye position, wide jawline, and high forehead.

## 5. Discussion

The present study used GPOR (Chu et al., 2005), a non-linear regression analysis, to estimate utility functions that describe the relationship between multivariate children's facial shape traits and the degree of perceived cuteness. We measured children's face shapes and constructed a face space through feature dimensionality reduction using a combination of methodologies of geometric morphometrics (Bookstein, 1991; Dryden and Mardia, 1998) and a PCA. Participants were instructed to rate the cuteness of the facial images synthesized within the face space on a 5-point scale. Furthermore, the average utility function was calculated, and the faces with the maximum and minimum values were synthesized.

### 5.1. Effectiveness of the proposed method

The results of a LOOCV of ordinal prediction accuracy showed that the GPOR model had better prediction accuracy than the logistic ordinal regression model, indicating that non-linear models are effective in estimating the utility function of perceived cuteness. Another advantage of using non-linear regression models is that it allows the estimation of face shapes with maximum and minimum perceived cuteness. Among non-linear regression models (e.g., LOESS model, Komori et al., 2009b), The GPR model is superior because it can assess prediction uncertainty.

The commonly used method for estimating multivariate utility functions is a technique similar to the analysis of variance, called conjoint analysis (Rao et al., 2014). However, the conjoint analysis generally does not examine higher-order interactions because it requires a more significant number of trials. Moreover, the complicated balance of facial components influences perceived cuteness. Thus, the GPR is more suitable for studies on faces than conjoint analysis.

One of the conventional methods used to examine the morphological characteristics of faces related to perceived cuteness is to create a composite image of face images judged to be relatively cute and explore the characteristics of the composite image (Sprengelmeyer et al., 2009; Lobmaier et al., 2010; Hahn et al., 2013; Nittono et al., 2022). Such methods implicitly assume that the cuteness utility function exhibits a unimodal response, but if this response were multimodal, such procedures could lead to erroneous conclusions. Contrastingly, using GPR, it is possible to examine the relationship between face shape and perceived cuteness even if the utility function is multimodal. The results of this study do not show that the utility function for cuteness was multimodal. Nevertheless, future studies may find multimodality in the estimated function when adding the facial feature dimension.

### 5.2. Facial features associated with perceived cuteness

The parameters corresponding to the maximum value of the mean utility function suggest that a face with a low eye position, small chin, narrow jawline, and a small forehead are features that enhance perceived cuteness. Almanza-Sepúlveda et al. (2018) examined the relationship between infants' facial traits and cuteness argued that small chin and narrow jawline are related to the roundness of the face, and consequently, the roundness leads to cuteness. The results of this study are consistent with this explanation.

Although many previous studies have argued that a large forehead is a cue for perceived cuteness (e.g., Hildebrandt and Fitzgerald, 1979), the results of this study suggest that forehead height itself does not contribute to cuteness enhancement. Rather, the width of forehead (represented in PC1) appears to be associated with perceived cuteness. Contrastingly, the relatively low position of facial parts such as the eyes and mouth contribute to cuteness. These results suggest that high foreheads may impair the roundness of the face, resulting in low cuteness. It should be noted that while previous studies examined infants' faces, this study used faces of 3- to 4-year-old children as stimuli. This discrepancy may be the cause of the inconsistency of the results. This means that different morphological factors may have affected perceived cuteness in the previous and present studies. It is necessary to conduct further research on the possibility that the morphological features of the face that affect perceived cuteness change along with growth.

### 5.3. Limitations and Future Directions

In this study, the locations of facial landmark coordinates were determined based on previous studies (Kamachi et al., 2001; Komori and Nittono, 2013), in which the shape of the forehead was measured with reference to the hairline. However, the shape of the hairline did not fully and accurately represent or replicate the features of the child's forehead. In the future, 3D measurements would be needed to encompass and replicate the three-dimensional shape of the forehead, which reflects the facial features of children.

Next, children's facial features were reduced to three PCs using PCA. Nonetheless, these three dimensions do not fully explain the variations in the facial features of young children. Notably, facial features not included in the three dimensions might affect perceived cuteness. Therefore, future studies should consider even greater dimensions of facial features. However, it is unclear how many trials would be required to construct a model with sufficient prediction accuracy when the number of dimensions considered was increased.

## 6. Conclusion

This study examined the relationship between children's face shapes and perceived cuteness in multivariate data using GPOR. Gaussian process ordinal regression is an extension of GPR that can be applied to ordinal scale responses and has not been used in psychological research before. This study, estimated the average utility function of perceived cuteness based on responses to a Likert scale, thereby identifying facial features associated with perceived cuteness.

This method can be applied to both research relating to facial cuteness and that pertaining to the study of various facial assessments, such as facial attractiveness, impressions, and stereotypes, and the shapes of the utility functions estimated from these investigations will provide clues to finding the factors that determine various judgments on faces.

## Data availability statement

The raw data supporting the conclusions of this article will be made available by the authors, without undue reservation.

## Ethics statement

The studies involving human participants were reviewed and approved by Behavioral Research Ethics Committee of the Osaka University School of Human Sciences (HB019-058) and the Ethics Committee of the Osaka Electro-Communication University (18-004). Written informed consent to participate in this study was provided by the participants' legal guardian/next of kin. Written informed consent for the publication of potentially identifiable images/data was not required as the figures included in the manuscript are composites generated using a large number of individual images.

## Author contributions

MK, KS, and HN contributed to the conception, designed the study, and performed the experiment. MK and HN collected, measured, and synthesized facial images. MK, TT, and KS implemented the experimental and analytical programs. Further, MK and TT performed the statistical analysis. Finally, MK and TT wrote the draft of the manuscript and made figures and tables. All authors contributed to manuscript revision and read and approved the submitted version.

## Funding

This study was supported by the JSPS KAKENHI (grant nos. 19K03375 to MK, 17H02651, and 21H04897 to HN).

## Conflict of interest

The authors declare that the research was conducted in the absence of any commercial or financial relationships that could be construed as a potential conflict of interest.

## Publisher's note

All claims expressed in this article are solely those of the authors and do not necessarily represent those of their affiliated organizations, or those of the publisher, the editors and the reviewers. Any product that may be evaluated in this article, or claim that may be made by its manufacturer, is not guaranteed or endorsed by the publisher.
